# Production of Activated Carbons from Food/Storage Waste

**DOI:** 10.3390/ma16041349

**Published:** 2023-02-05

**Authors:** Małgorzata Wiśniewska, Natalia Pawlak, Dariusz Sternik, Robert Pietrzak, Piotr Nowicki

**Affiliations:** 1 Department of Radiochemistry and Environmental Chemistry, Institute of Chemical Sciences, Faculty of Chemistry, Maria Curie-Sklodowska University in Lublin, M. Curie-Sklodowska Sq. 3, 20-031 Lublin, Poland; 2 Department of Applied Chemistry, Faculty of Chemistry, Adam Mickiewicz University in Poznań, Uniwersytetu Poznańskiego 8, 61-614 Poznań, Poland; 3 Department of Physical Chemistry, Institute of Chemical Sciences, Faculty of Chemistry, Maria Curie-Sklodowska University in Lublin, M. Curie-Sklodowska Sq. 3, 20-031 Lublin, Poland

**Keywords:** activated carbons, chemical activation, waste biomass utilization, microwave heating, adsorption, physicochemical properties

## Abstract

This paper deals with the adsorption of organic and inorganic pollutants on the surface of carbonaceous adsorbents prepared via the chemical activation of expired or broken food products—the solid residue of the “cola-type” drink as well as spoilt grains of white rice and buckwheat groats. The activation process was conducted in the microwave furnace with the use of two activating agents of different chemical nature—potassium carbonate and orthophosphoric acid. The activated carbons were characterized based on the results of elemental analysis, low-temperature nitrogen adsorption/desorption, Boehm titration, thermal analysis, and scanning electron microscopy. Additionally, the suitability of the materials prepared as the adsorbents of methylene blue and iodine from the aqueous solutions was estimated. The materials obtained via chemical activation with H_3_PO_4_ turned out to be much more effective in terms of both model pollutant adsorptions. The maximum sorption capacity toward iodine (1180 mg/g) was found for the white-rice-based activated carbon, whereas the most effective in the methylene blue removal (221.3 mg/g) was the sample obtained from the solid residue of the expired “cola-type” drink. For all carbonaceous materials, a better fit for the experimental adsorption data was obtained with the Langmuir isotherm model than the Freundlich one.

## 1. Introduction

Adsorption is an exothermic process associated with mass transfer that leads to molecule attraction by the solid surface. Due to unbalanced forces, atoms on the solid surface have residual energy, so adsorbate molecules or ions form the interface layer. This results in their higher concentration on the surface than in the bulk solution [1,2]. This process is usually described at the equilibrium state based on the isotherms and kinetic and thermodynamic studies [3,4,5]. Additionally, characterization of the adsorbent, using, for example, FTIR, SEM, TEM, XRD, and XPS analysis before and after the formation of the adsorption layer, provides important information about the mechanisms of this phenomenon [6,7,8]. In many systems, the adsorption mechanism is complex and dependent on many factors relating to the solid, adsorbate, and solution properties [9,10].

Adsorption is widely applied in the pharmaceutical, cosmetic, textile, food, and paint industries as well as in water or wastewater treatment and air purification [11,12,13]. Most heterogeneous catalytic reactions proceed through the adsorption of the gaseous reactant on the solid catalyst increasing its concentration and, consequently, the rate of reaction [14]. It plays a fundamental role in ecology due to the regulation of the exchanges between the geosphere, hydrosphere, and atmosphere, the transport of substances in the ecosystems, and the occurrence of enzymatic processes [15,16].

Various materials can be used in the role of adsorbents. They include activated carbons, zeolites, minerals, metal oxides, hydroxide-based adsorbents, resins, as well as composite adsorbents [17,18,19,20,21]. However, in wastewater treatment, activated carbons demonstrate a favorable relationship between their adsorption capacity and the cost of production. They are obtained by physical or chemical activation of almost all carbonaceous organic materials, such as coal, peat, wood, coconut shells, and different kinds of biomass, for example, straw, corncobs, fruit stones, fruit peels, walnut shells, or wood sawdust [19,22,23,24,25,26]. In the case of biomass, which is very often a cumbersome waste material, this leads to its management and reuse as well as the elimination of greenhouse gas production as a result of its putrefaction [27]. The activation process of these materials (especially via the chemical method) leads to the formation of microporous or mesoporous structures, large specific surface area, as well as a specific composition of surface oxygen groups. For this reason, the activated carbons exhibit excellent adsorption affinity for most pollutants present in the liquid phase (for example, heavy metal ions, dyes, drugs, pesticides, phenols, amines, polymers, surfactants) [28,29] as well as in the gas phase (i.e., nitrogen and sulfur oxides, carbon dioxide, hydrogen sulfide, volatile organic compounds, etc.) [30,31].

The main purpose of this paper was to produce a series of activated carbons by means of chemical activation of food products intended for disposal and estimation of the suitability of carbonaceous materials obtained in this way in terms of removing organic and inorganic impurities from the aqueous environment. As a part of the ecological approach to producing carbonaceous adsorbents, waste materials containing carbon in organic combinations were used as precursors, such as the expired “cola-type” drink, white rice, and buckwheat groats affected by pests. The basic activator, potassium carbonate, and an acidic activator, orthophosphoric acid, were used in order to activate the above-mentioned precursors. Microwave heating was applied during the adsorbent synthesis. This heating variant has many advantages over the conventional one, which is most often used for the production of carbon adsorbents. The main advantage of thermal treatment using microwave energy is the rapid and uniform heating of the material throughout the whole sample volume, which allows for better control of the parameters of the activation procedure as well as shortening its time and reducing the final temperature of the process. In addition to the basic physicochemical characteristics of the obtained carbon materials (including the determination of their elemental composition, the chemical nature of the surface, and basic textural parameters), adsorption tests were carried out against the two model pollutants—iodine and methylene blue. In the case of the organic dye, the effect of its initial concentration on the efficiency of adsorption from the aqueous solution was investigated.

## 2. Materials and Methods

### 2.1. Materials

The precursors of the activated carbons were: the solid residue of expired “cola-type” drink (CD), white rice (WR), and buckwheat groats (BG) affected by pests. Prior to the activation, the expired cola drink was dried at 50 °C in order to evaporate the water. The other two precursors were not subjected to any pre-treatment. All of the materials came from the store warehouses in the Wielkopolska region (Poland). For each of the starting materials, two variants of the activation procedure were applied: (1) chemical activation with orthophosphoric acid at 600 °C (AH) and (2) chemical activation with potassium carbonate at 600 °C (AK). In the beginning, the raw materials were impregnated with 50% H_3_PO_4_ (POCH, Gliwice, Poland) or water solution of K_2_CO_3_ (POCH, Gliwice, Poland) at the precursor—activating factor weight ratio equal to 1:1. After the impregnation step (24 h, room temperature) the samples were dried at 110 °C to complete evaporation of water, placed into the quartz crucibles and heated in the nitrogen atmosphere (flow rate 250 cm^3^/min) in the microwave muffle furnace (Phoenix model) provided by CEM Corporation (Matthews, IL, USA). In the first stage, the samples were heated to 200 °C at the heating rate of 5 °C/min and annealed at that temperature for 30 min. In the next stage, the samples were heated to the final activation temperature of 600 °C (at the heating rate of 5 °C/min) and kept at that temperature for 30 min. After that time, the samples were cooled down to room temperature under the nitrogen flow. The samples activated with potassium carbonate were subjected to the two-step washing procedure, firstly with 0.5 dm^3^ of hot 5% solution of hydrochloric acid (POCH, Gliwice, Poland) and next with 10 dm^3^ of hot distilled water until it was free from chloride ions. Finally, the activated carbons were dried at 110 °C to the constant mass and labeled CDAK, WRAK, and BGAK, respectively. In the case of the samples activated with H_3_PO_4,_ the one-step washing procedure was applied. The solids were washed with 10 dm^3^ of hot distilled water using a vacuum filtration funnel (with a glass sintered disc) and dried to the constant mass at 110 °C. The activated carbons obtained in this way were designated CDAH, WRAH, and BGAH, respectively.

### 2.2. Analytical Procedures

The elemental analysis of the activated carbons was performed using the CHNS Vario EL III elemental analyzer provided by Elementar Analysensysteme GmbH (Langenselbold, Germany). The total content of mineral matter (ash) was evaluated according to the ISO 1171:2002 Standard. In order to characterize the chemical nature of the activated carbon surfaces, the pH value of their aqueous extracts was determined in accordance with the procedure described in [30]. The content of the surface functional groups of basic or acidic character was determined according to the Boehm titration method, described in detail in our previous paper [32]. The textural characterization of the carbonaceous materials was based on the nitrogen adsorption–desorption isotherms measured at –196 °C using the sorptometer Autosorb iQ, provided by Quantachrome Instruments (Boynton Beach, FL, USA). Prior to the analysis, the activated carbon samples were degassed under vacuum at 300 °C for 12 h in order to remove all pre-adsorbed gaseous species. The specific surface area of the materials was determined on the basis of the multilayer adsorption BET (Brunner–Emmett–Teller) theory. The pore size distribution, as well as the total pore volume for each activated carbon sample, were determined based on the BJH (Barrett–Joyner–Halenda) model. The surface morphologies of the samples were observed by scanning electron microscopy (SEM) with the Quanta 250 FEG instrument provided by FEI (Waltham, MA, USA) equipped with the detector Octane Elect Plus provided by EDAX (Berwyn, IL, USA). Thermal properties of the activated carbons were determined using the simultaneous thermal analyzer STA 449 F1 Jupiter (Netzsch, Selb, Germany) under the following operational conditions: the heating rate of 10 °C/min, the dynamic atmosphere of helium (50 cm^3^/min) in the temperature range of 30–1200 °C, the sample mass of about 23 mg, the sensor thermocouple type P TG-DSC. As a reference, the empty Al_2_O_3_ crucible was used. The gaseous products emitted during the decomposition of the carbonaceous materials were analyzed by the quadrupole mass spectrometer QMS 403D Aëolos (Netzsch, Selb, Germany) coupled online to the thermal analyzer.

### 2.3. Adsorption Experiments

In order to determine the sorption capacity of the obtained activated carbons, adsorption tests were carried out against the two model impurities, i.e., the aqueous solution of iodine (representing inorganic pollutants with small molecule sizes—close to 1 nm) and the aqueous solution of methylene blue (a cationic dye, representing organic heterocyclic aromatic pollutants).

The iodine sorption ability of the activated carbons was determined according to the procedure described in detail in the PN-83/C-97555.04 National Standard. Briefly: 0.2 g of the carbon sample was placed in 150 cm^3^ flasks and mixed with 4 cm^3^ of 5% HCl (POCH, Gliwice, Poland) and 20 cm^3^ of 0.1 mol/dm^3^ iodine solution (POCH, Gliwice, Poland). The mixture was shaken for 4 min, filtered through the filter paper, and washed with 50 cm^3^ of distilled water. The resulting solution was titrated with 0.1 mol/dm^3^ sodium thiosulphate (POCH, Gliwice, Poland).

The study of the sorption abilities of the activated carbons toward methylene blue (MB) was carried out according to the following procedure: the samples of the prepared activated carbons in the same portions of 0.025 g (particle size below 0.1 mm) were added to 50 cm^3^ of the MB aqueous solutions (POCH, Gliwice, Poland) with the initial concentrations ranging from 5 to 200 mg/dm^3^ and then the suspensions were stirred magnetically (150 rpm) at the temperature of 22 ± 1 °C for 12 h to reach the equilibrium state. After that time, the suspensions were centrifuged at 5000 rpm for 10 min, using the Frontier ™ centrifuge FC5515 provided by OHAUS (Parsippany, NJ, USA) to eliminate the activated carbon particles from the analytes intended for spectroscopic measurements. The dye concentrations in the solution before and after the adsorption tests were established by applying the double beam UV–Vis spectrophotometer Cary 100 Bio provided by Agilent (Santa Clara, CA, USA) at the wavelength of 665 nm using the previously prepared calibration curve. Distilled water was applied as the reference sample.

The amount of methylene blue adsorbed in the equilibrium state (*q_exp_*, mg/g) was calculated from the following Equation (1):

(1) $$q_{exp}=\frac{\Delta c\cdot V_{MB}}{m_{AC}}$$

where Δ*c*—the difference between the initial and final concentrations of the methylene blue [mg/dm^3^]; *V_MB_*—the volume of the dye solution [dm^3^]; *m_AC_*—the mass of the activated carbon used during the adsorption test [g].

The effectiveness of methylene blue adsorption (*E_ads_*, %) from the aqueous solutions was calculated according to Equation (2):

(2) $$E_{ads}=\frac{\Delta c}{c_{init}}\cdot 100\%$$

where *c_init_*—the initial methylene blue concentration [mg/dm^3^].

For the modeling of adsorption isotherms, we used the Langmuir (3) and Freundlich (4) equations:

(3) $$q_{eq}=\frac{q_{m}K_{L}c_{eq}}{1+K_{L}c_{eq}}$$

(4) $$q_{eq}=K_{F}c_{eq}^{1/n}$$

where *q_eq_*—the equilibrium MB adsorbed amount [mg/g]; *q_m_*—the maximal MB adsorbed amount (monolayer capacity) [mg/g]; *K_L_*—the Langmuir constant [dm^3^/mg]; *c_eq_*—the equilibrium concentration of MB [mg/dm^3^]; *K_F_*—the Freundlich constant [mg/g (mg/dm^3^)^1/n^]; *n*—the Freundlich parameter.

## 3. Results and Discussion

### 3.1. Elemental Composition of the Prepared Activated Biocarbons

All activated carbons prepared via microwave-assisted chemical activation with orthophosphoric acid and potassium carbonate were first subjected to the elemental analysis, the results of which are presented in Table 1.

The obtained data clearly show that both the kind of precursor and the type of activating agent applied during activation have a significant impact on the ash content in the structure of the activated carbons. The largest ash content (6.9% by weight) was found for the BGAH sample obtained by the activation of buckwheat groats with H_3_PO_4_. An equally large proportion of the mineral substance was observed for the analogous WRAH-activated carbon prepared from white rice. However, in the case of the CDAH sample obtained in the same way from the residue of a “cola-type” drink, the ash contribution is almost two-fold smaller (3.5 wt.%). These differences result from the diverse origin of the individual precursors. As is well known, groats and rice are produced from cereal grains that are not subjected to intensive thermochemical treatment, the result of which is that the content of mineral admixtures in their structure is quite large and depends on the conditions of cereal cultivation to a great extent. The “cola-type” drink residue is mostly sugar, obtained through the complex thermochemical treatment of sugar beet or sugar cane, which makes the share of mineral admixtures significantly smaller.

As follows from the further analysis of the data presented in Table 1, the mineral content in the structure of the activation products is largely determined by the activation and post-treatment procedure. In the case of the activated carbons obtained with potassium carbonate, the ash content is significantly smaller than for the analogous samples obtained by the activation with orthophosphoric acid. This can be a consequence of the different chemical nature of both activating agents (basic or acidic) as well as the mechanism of their interactions with the precursors. However, the procedure of washing the activation process products also has a great influence. As mentioned earlier, the two-step washing procedure was applied for the carbonate-activated samples. Hot hydrochloric acid used in the first stage of this procedure could contribute to a significant reduction in the content of mineral substances in the structure of the final products.

According to the data presented in Table 1, the activated carbons prepared from expired or broken food products also differ significantly in terms of the content of elemental carbon as well as heteroatoms, such as nitrogen, sulfur, and oxygen. The greatest contribution of carbon (83.4 and 82.5 wt. %) is found for the samples prepared via chemical activation of the solid residue of the expired “cola-type” drink. These samples are also characterized by the smallest share of oxygen and other non-carbon admixtures in the structure. The contribution of individual elements in the activated carbon structure is also conditioned by the type of the activating agent. The samples activated with H_3_PO_4_ are characterized by a larger content of elemental carbon and hydrogen than analogous materials activated with K_2_CO_3_. On the other hand, in the case of nitrogen and oxygen, the opposite relationship was observed. This is particularly evident for the WRAK and WRAH samples. Moreover, according to the results of the SEM-EDX analysis, the CDAH and WRAH samples (obtained via the chemical activation with phosphoric acid) also contain some amounts of phosphorus in their structure—that is, 3.14 and 1.65 wt. %, respectively. This is probably a consequence of the phosphate group incorporation into the carbon matrix.

### 3.2. Acidic—Basic Properties of the Activated Carbons Prepared from Waste Food Products

In order to fully characterize the acidic-basic properties of the obtained activated carbons, the content of surface functional groups was determined, and the pH values of their aqueous extracts were measured. According to the data presented in Table 2, the carbonaceous materials differ significantly in terms of the chemical character of the surface. All carbons activated with H_3_PO_4_ show a strongly acidic character, and their pH value ranges from 3.5 to 4.0, whereas the analogous samples activated by potassium carbonate (especially the WRAK sample) show a slightly acidic or almost neutral character of the surface (pH value is in the range 5.3–6.7). The type of precursor used for activated carbons production has a definitely smaller impact on their acidic-basic properties. The samples obtained from the solid residue of the “cola-type” drink, as well as buckwheat groats, are characterized by the very similar nature of the surface. Only in the case of the WRAH and WRAK-activated carbons (obtained as a result of white rice activation), significantly higher pH values were noted.

The activated carbons obtained from the waste food products also differ in terms of the content and type of functional groups. Each of the tested samples contains functional groups of both acidic and basic nature (with a clear predominance of the former); however, their mutual ratio changes depending on the type of the precursor and the method of its activation. The largest total content of surface groups (1.78 mmol/g) is exhibited by the WRAK sample, obtained as a result of the microwave-assisted K_2_CO_3_ activation of white rice. This sample is also characterized by the greatest share of acidic and basic groups among all the carbon materials under investigation. The least functionalized surface (1.16 mmol/g) is found in the analogous sample WRAH, activated by orthophosphoric acid. Interestingly, a similar dependency was noted for both of the other precursors. The samples activated with potassium carbonate (CDAK and BGAK) contain more acidic and basic functional species on their surface than the analogous materials obtained via activation with H_3_PO_4_. This observation points out a different mechanism of interactions between the individual activating agents and the starting materials used in this study. In the case of the amount of basic functional groups, the influence of the precursor type is also visible. The activated carbons obtained from the rice grains contain three-fold more groups of this kind than the analogous carbonaceous materials obtained from the solid residue of the “cola-type” drink.

### 3.3. Textural Parameters of the Activated Carbons Prepared from Waste Food Products

The most important physicochemical parameters of the activated carbons (especially from the adsorption point of view) are the size of the specific surface area and the total pore volume, as well as the pore size distribution. According to the data summarized in Table 3, all the activated carbons obtained via the microwave-assisted chemical activation of waste food products have a well-developed surface area and a porous structure composed of significant amounts of micropores and mesopores. The experimental results also showed that the textural parameters of the activated carbons depend significantly on the type of precursor and the activating agent used for their production.

The most developed surface area (1033 m^2^/g) was found in the WRAH sample, obtained via the chemical activation of waste white rice with H_3_PO_4_, whereas the least favorable in this respect (S_BET_ only 318 m^2^/g) was the CDAK sample, prepared by means of the activation of the solid residue of the “cola-type” drink with potassium carbonate. However, it should be emphasized that the results obtained for the CDAK and especially for CDAH samples are definitely better than for nitrogen-enriched activated carbon obtained by physical activation of solid residues of waste sweet drinks (surface area 198 m^2^/g, total pore volume 0.157 cm^3^/g) [33]. The data collected in Table 3 clearly show that the carbons activated with orthophosphoric acid (regardless of the type of precursor) are characterized by much better textural parameters than the analogous materials obtained with K_2_CO_3_. This is particularly evident for the carbons obtained from the solid residue of the “cola-type” drink, where the surface area of the acid-activated sample (CDAH) is more than three times larger than that of the alkali-activated carbon (CDAK). This fact confirms the different reaction mechanisms between the precursors of activated carbons and activating agents of Lewis acid (H_3_PO_4_) and Lewis base (K_2_CO_3_) character [34]. The probable reason for less favorable textural parameters of the samples activated with potassium carbonate can also be overly mild conditions of the activation process (temperature 600 °C, time 30 min, and precursor–activator weight ratio of 1:1). Typically, this variant of activation is carried out at higher temperatures and with an excess of an activating agent.

**Figure 1:**
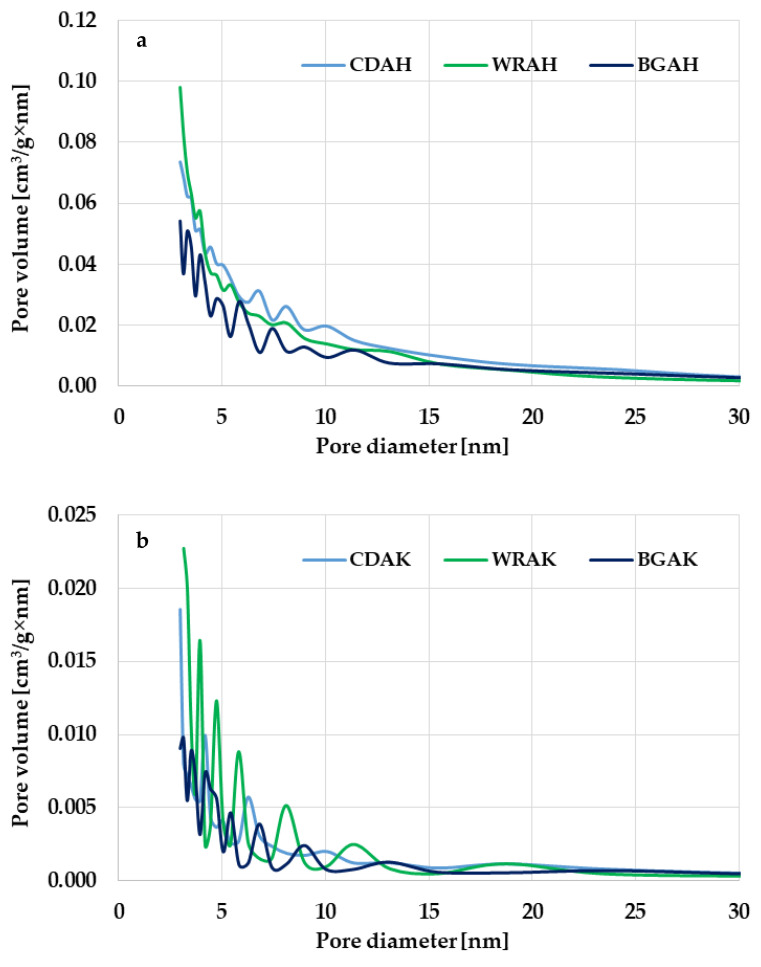
Pore size distribution of the samples activated with H_3_PO_4_ (**a**) and K_2_CO_3_ (**b**).

Based on the analysis of the data presented in Table 3, it can be concluded that the heat treatment of expired or broken food products in the presence of potassium carbonate or orthophosphoric acid leads to activated carbons with a micro-mesoporous structure. The micropore contribution to the total pore volume depends, first of all, on the type of the activating agent. The influence of the precursor type is less pronounced. The greatest contribution of micropores of 69% is found in the WRAK sample obtained by the chemical activation of waste white rice with K_2_CO_3_. In the case of the second activated carbon obtained from the same precursor but via the activation with H_3_PO_4_ (WRAH sample), the proportion of micropores is smaller by 17%. The same dependency is observed for the other precursors. Carbons obtained with the use of the basic activating agent are characterized by a significantly greater share of micropores in the structure than the analogous materials subjected to the thermal treatment in the presence of the activating agent of acidic character. The greatest difference in this respect (21%) was noted for the samples obtained from buckwheat groats. The confirmation of these observations is the course of the pore size distribution curves shown in Figure 1. The products of microwave-assisted chemical activation with H_3_PO_4_ have a great volume of pores with a diameter in the range of 2–25 nm, including small and medium mesopores (Figure 1a). In the case of the analogous activated carbons obtained via the chemical activation with potassium carbonate (Figure 1b), the pore volume is significantly smaller; however, the pore size distribution is much narrower and ranges from 2 to 15 nm.

**Figure 2:**
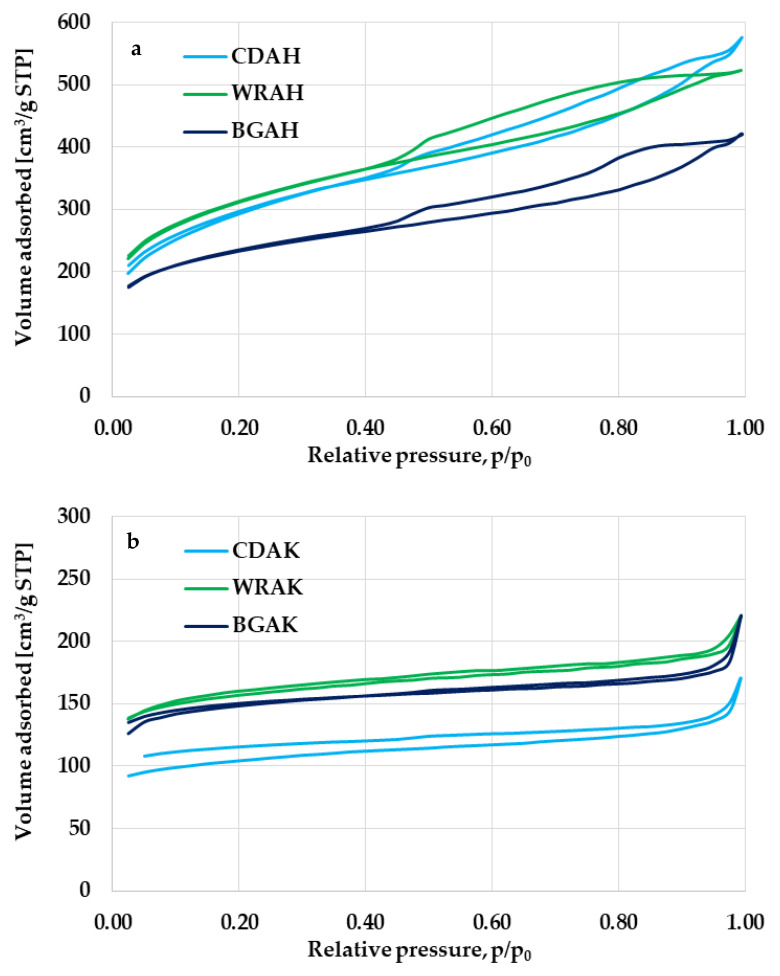
Low-temperature nitrogen adsorption/desorption isotherms of the samples activated with H_3_PO_4_ (**a**) and K_2_CO_3_ (**b**).

Significant differences between the porous structure of carbons activated with H_3_PO_4_ and K_2_CO_3_ are also confirmed by the courses of low-temperature nitrogen sorption isotherms presented in Figure 2. The shape of the N_2_ adsorption/desorption isotherms observed for the CDAH, WRAH, and BGAH samples (Figure 2a) is similar to the type IV according to the IUPAC classification. The characteristic and very broad hysteresis loop (close to the H4 type) indicates the presence of a significant amount of mesopores in the structure of the activated carbons prepared via activation with orthophosphoric acid. In turn, in the case of the corresponding samples activated with potassium carbonate, the shape of N_2_ sorption isotherms is similar to type I according to the IUPAC classification, which means that these carbonaceous materials contain considerable amounts of micropores and small mesopores in their structure.

**Figure 3:**
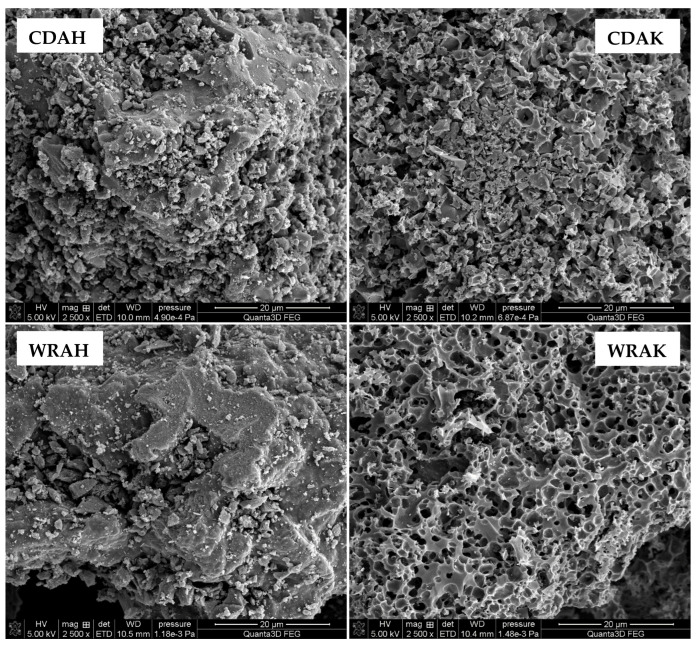
SEM images of the selected samples activated with H_3_PO_4_ and K_2_CO_3_.

Textural and morphological diversities of individually activated carbons are also confirmed by the SEM images presented in Figure 3. Depending on the precursor type and the activation procedure, the samples differ significantly in terms of the number, shape, and size, as well as the arrangement of pores, holes, and slits. These differences are particularly well seen for the samples obtained by the microwave-assisted chemical activation of white rice grains. In the case of the sample activated with potassium carbonate (WRAK), numerous pores of various diameters and almost circular shapes are observed. In addition, many of these pores are interconnected. In the case of the carbonaceous material activated with orthophosphoric acid, the number of pores is smaller, whereas their diameter is considerably larger. Unfortunately, a significant part of the pores is blocked by small particles, which can be both very small pieces of activated carbon grains (carbon dust) and the mineral substance (ash)—the brighter fragments observed in the SEM images. In the case of the analogous materials obtained via the chemical activation of the solid residue of the “cola-type” drink, the situation is quite similar.

### 3.4. Thermal Properties of the Activated Carbons Prepared from Waste Food Products

**Figure 4:**
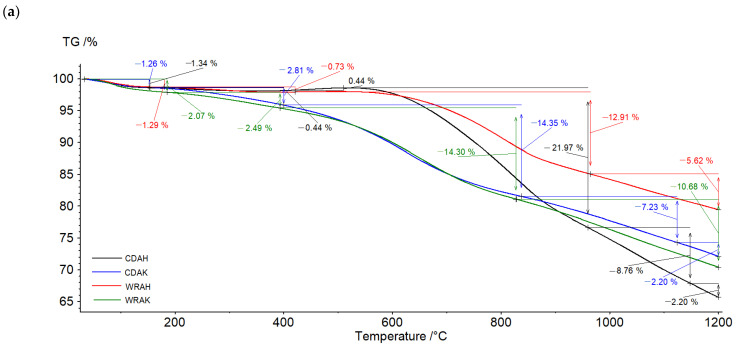

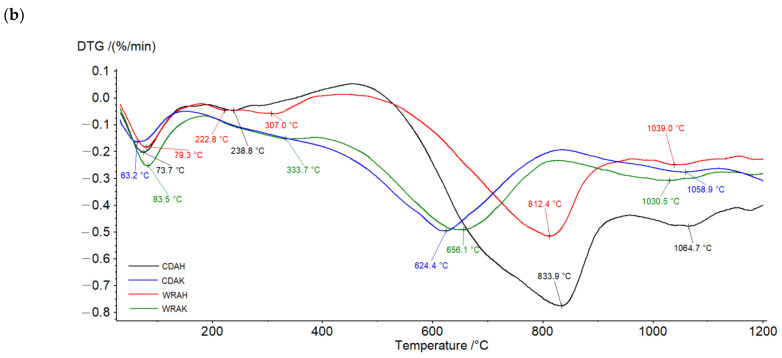
TG (**a**) and DTG (**b**) curves of the activated carbons obtained in the helium atmosphere.

**Figure 5:**
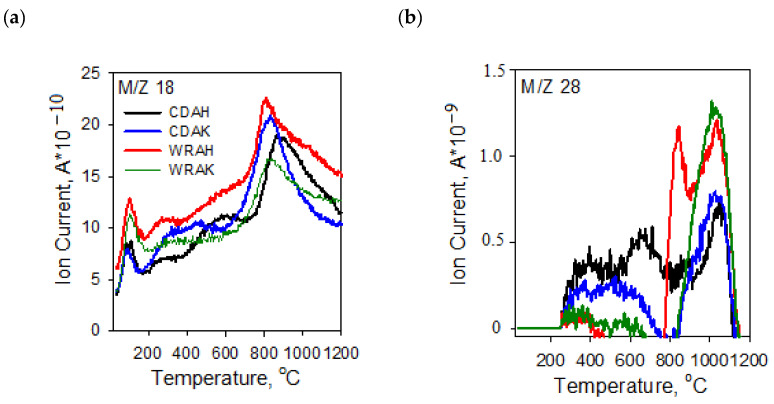

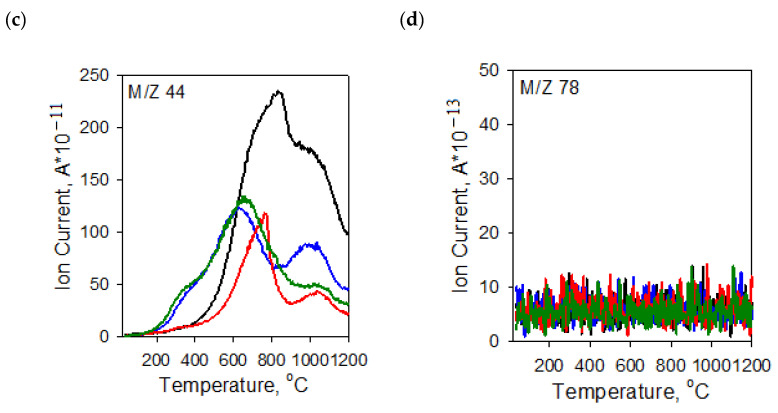
MS profiles of gaseous products of the activated carbons structure defragmentation: (**a**) *m/z* 18 (H_2_O; (**b**) *m/z* 28 (CO); (**c**) *m/z* 44 (CO_2_); (**d**) *m/z* 78 (C_6_H_6_) obtained in the helium atmosphere.

The character of TG and DTG curves presented in Figure 4 indicates that the activated carbons show different thermal stability, dependent mainly on the procedure of activation. Irrespective of the precursor type, the samples activated with potassium carbonate are characterized by smaller thermal stability than the analogous materials subjected to activation in the orthophosphoric acid presence. As reflected by the TG and DTG curves, the process of thermal decomposition of the activated carbon structures occurs in several stages. The mass losses change in the range from 1.26% (for the CDAK sample) to 2.07% (for the WRAK sample, Figure 4a), and the corresponding peaks on the DTG curves (Figure 4b) are related to the endothermic process of physically adsorbed water removal (dehydration process), which is clearly visible in the MS spectra characteristic of water (Figure 5a, *m/z* 18). At higher temperatures, the pyrolysis process is associated with the decomposition of oxygen functional groups present on the activated carbon surfaces. In the temperature range of up to 400 °C, the observed mass losses are the smallest for the CDAH (0.44%) and WRAH (0.73%) samples. In the case of these samples, an increase in the signal for CO_2_ was also recorded (Figure 5c, *m/z* 44), which was most probably related to the decomposition of carboxyl groups. For the remaining two activated carbons, the observed mass losses were much greater (2.81% for the CDAK and 2.49% for WRAK samples) with the simultaneous release of CO (Figure 5b) and CO_2_ (Figure 5c). This indicates the thermal decomposition of carboxyl groups (CO_2_ emission), carboxylic anhydrides (CO and CO_2_ emission), as well as lactone groups at higher temperatures (CO_2_ emission). Mass losses greater than 14%, recorded for the CDAK and WRAK samples at about 810 °C are mainly related to the emission of CO_2_ and smaller amounts of CO, which can indicate the decomposition of surface lactone groups (CO_2_ emission) and carboxylic anhydrides (CO_2_ and CO emission). In the case of the other examined activated carbons, clear peaks on the DTG curves are observed at higher temperatures, i.e., at 834 °C (with a mass loss of 22%) for the CDAH and at 812 °C (mass loss of 13%) for the WRAH samples. They are related to the emission of CO_2_, CO, and H_2_O, resulting most probably from the decomposition of phenolic (CO), ether (CO), and carbonyl (CO) groups, as well as pyrone-like structures (CO_2_, CO). Above 900 °C, further decomposition of pyrone-like structures takes place. The lack of peaks in Figure 5d (presenting the *m/z* 78 ion emission) indicates that no benzene derivatives were found in the gaseous products of decomposition, which could mean that there are no aromatic groups on the activated carbon surfaces.

### 3.5. Adsorption Properties of the Activated Carbons Prepared from Waste Food Products

**Figure 6:**
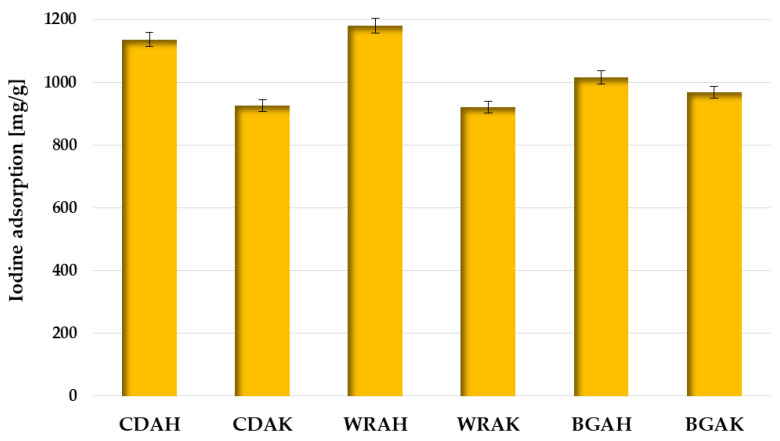
Iodine adsorption by the activated carbons prepared from waste food products.

In order to test the sorption abilities of the obtained activated carbons and assess their usefulness for the removal of impurities with a small molecule size from the liquid phase, the iodine number was determined. According to the data presented in Figure 6, carbon materials obtained as a result of activation with orthophosphoric acid are much better in terms of iodine adsorption. This is particularly visible for the WRAH and WRAK samples obtained from white rice grains, where the difference in the sorption capacity is as great as 259 mg/g.

This is most likely a consequence of the much more developed specific surface area and porous structure of these samples (Table 3), which favors the adsorption of this type of pollutant. The effectiveness of iodine removal from the aqueous solutions is also conditioned by the type of precursor used for the activated carbon production, but the impact of this parameter is considerably smaller.

Based on the analysis of the data presented in Table 4, it can be concluded that the WRAH and CDAH samples perform well in terms of iodine adsorption when compared to the carbonaceous materials obtained from the other waste materials or commercial products.

The sorption capacity of both activated carbons toward iodine is significantly smaller in comparison with activated carbon prepared by chemical activation of prickly pear seed cake with H_3_PO_4_. Dhahri et al. proved that this material is able to adsorb 2527 mg of iodine per g of adsorbent [34]. Slightly larger capacities were also obtained for the activated carbons prepared by steam activation of walnut shells (1450 mg/g) [35] and the activation of shea butter husk with potassium chloride (1244 mg/g) [36]. However, it should be emphasized that the activated carbons obtained from expired or broken food products perform better than adsorbents obtained from such precursors as spinach leaves, citrus fruit skins, olive stones, and the commercial products obtained from the non-renewable precursors, such as peat and bituminous coal [38,39,40].

**Figure 7:**
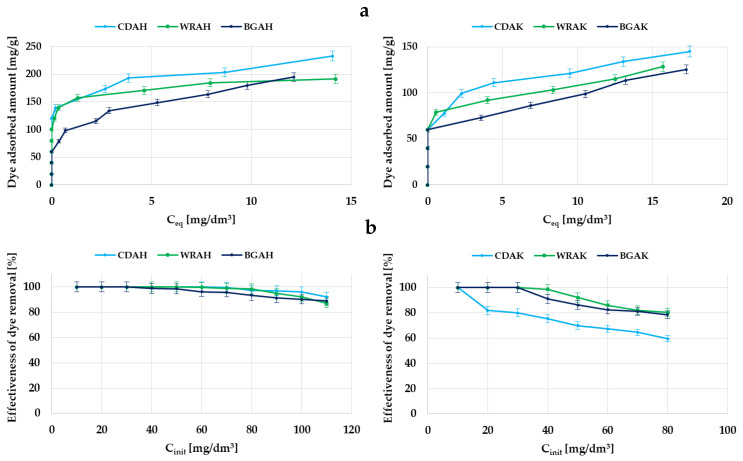
Adsorption of methylene blue on the samples activated with H_3_PO_4_ and K_2_CO_3_: the equilibrium adsorption of dye molecules as a function of its equilibrium concentration (**a**); the effectiveness of dye removal from aqueous solutions (**b**).

**Figure 8:**
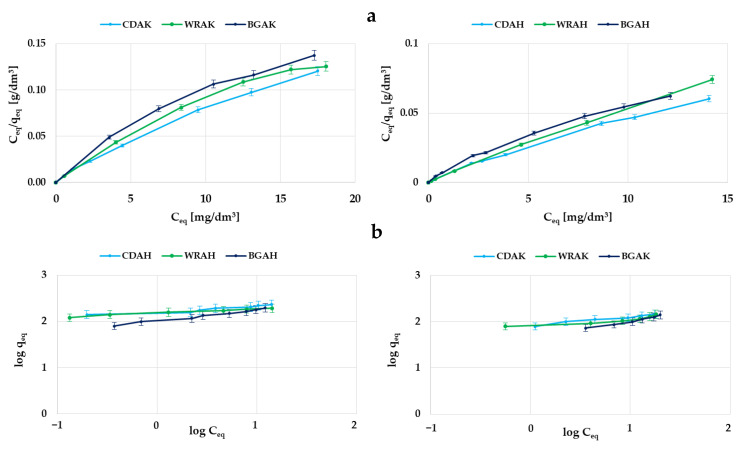
Adsorption of methylene blue on the samples activated with H_3_PO_4_ and K_2_CO_3_: the Langmuir isotherm plots (**a**); the Freundlich isotherm plots (**b**).

The second method of testing the sorption properties of the activated carbons prepared from waste food products was the assessment of the ability to remove organic pollutants from aqueous solutions based on the adsorption of the synthetic thiazine dye—methylene blue. The results of the adsorption tests are presented in Table 5 and Figure 7 and Figure 8.

According to the data summarized in Table 5, it turned out that the most effective methylene blue adsorbents were the carbons obtained as a result of the activation with orthophosphoric acid, especially the CDAH sample, which was able to adsorb 221.3 mg of dye per gram of the adsorbent. The sorption capacity of the analogous material produced with the use of potassium carbonate is more than two times lower (107.5 mg/g). In the case of the other two precursors, the differences between the samples activated with K_2_CO_3_ and H_3_PO_4_ are much smaller and do not exceed 60 mg/g. Significantly greater adsorption efficiency in the case of the carbons activated with the acidic agent is most probably the consequence of a much more developed specific surface area and greater contribution of mesopores in their structure. The presence of this type of pores is conducive to the effective adsorption of impurities with a larger molecular size, for example, organic dyes. This assumption is confirmed by the fact that the largest sorption capacity was obtained in the case of the CDAH-activated carbon, whose porous structure consists of mesopores in 60% of the cases. A significantly lower adsorption capacity toward methylene blue, observed in the case of the materials activated with potassium carbonate, can also be a consequence of the unfavorable influence of surface functional groups. These samples contain much more functional species of the acidic and basic character (Table 2), which in conjunction with the smaller pore diameter (Table 3), makes it difficult for the dye molecules to access the inside part of the pores.

As follows from the analysis of the data presented in Figure 7b, the majority of the samples activated with H_3_PO_4_ show 100% efficiency in the methylene blue removal in a wide range of its initial concentrations, i.e., from 10 to 60 mg/dm^3^. Importantly, even at concentrations of the order of 110 mg/dm^3^, the adsorption efficiency is very great and reaches about 90%. Unfortunately, in the case of the analogous materials activated with K_2_CO_3_ (in particular, the CDAK sample), the efficiency of synthetic thiazine dye removal from the aqueous solution is definitely smaller.

In order to determine the mechanism of dye molecule interactions with the obtained carbon adsorbents, the experimental data were analyzed using two popular models of adsorption—the Langmuir and Freundlich ones. The data summarized in Table 5 show that for all the activated carbons, the higher values of the correlation coefficient were obtained for the Langmuir model. This fact indicates that the methylene blue adsorption most probably proceeds with the formation of the adsorbate monolayer on the surface of the tested adsorbents. This is especially evident for the CDAH and WRAH samples, for which the value of determination coefficient R^2^ is close to 1. However, in the case of the WRAH and BGAH samples, equally high values of R^2^ were obtained for the Freundlich model; therefore, a slightly more complicated mechanism of adsorption—i.e., with the formation of a multilayer of adsorbate molecules on the surface of the obtained activated carbons—cannot be ruled out.

As follows from the analysis of the data collected in Table 6, the activated carbons prepared via the microwave-assisted chemical activation of waste food products perform quite well in terms of methylene blue adsorption when compared to the carbonaceous adsorbents obtained from various kinds of waste materials. The sorption capacity of the CDAH sample (the most effective among those obtained in this study—221 mg/g) is not as large as in the case of the activated carbons obtained from winemaking wastes, such as bagasse and cluster stalks (714–847 mg/g) [40] or the magnetic activated carbon obtained from expired beverage (405 mg/g) [41]. However, it should be emphasized that these materials were activated using a more reactive activating agent, i.e., KOH, with a higher weight ratio of reactants and at a higher temperature of thermochemical treatment. Importantly, most of the activated carbons obtained from expired or broken food products are characterized by a greater efficiency of methylene blue removal than the commercial activated carbon obtained from peat.

## 4. Conclusions

The studies proved that food waste could be successfully used as the precursor for the production of activated carbons. This could be a perfect and pro-ecological solution to the problem of waste management. It was shown that obtaining effective carbonaceous adsorbents requires an individual selection of the production procedure for each of the precursors. As follows, the use of microwave heating and two chemically different activating agents (K_2_CO_3_ and H_3_PO_4_) allows to prepare a wide gamut of activated carbons characterized by diverse physicochemical and adsorption properties. The food waste activation with H_3_PO_4_ leads to the adsorbents of acidic nature of the surface and well-developed mesoporous structure, which facilitates the diffusion of adsorbate molecules. Such prepared carbonaceous materials proved to be very effective in the inorganic and organic pollutants removal from aqueous solutions. In this respect, the most advantageous are the activated carbons obtained from the white rice grains and the solid residue of the “cola-type” drink. Their sorption capacities toward model impurities—iodine and methylene blue, assume the values of 1180 mg/g and 221.3 mg/g, respectively. Therefore, future research should be mainly focused on optimizing the procedure for producing this type of adsorbents.

## Figures and Tables

**Table 1:** Elemental composition of the activated carbons prepared via the chemical activation with H_3_PO_4_ and K_2_CO_3_.

Sample	Ash	Carbon ^1^	Hydrogen ^1^	Nitrogen ^1^	Sulphur ^1^	Oxygen ^1,2^
CDAH	3.5	83.4	2.6	0.0	0.0	14.0
CDAK	1.4	82.5	2.2	0.1	0.0	15.3
WRAH	6.8	80.4	2.7	0.8	0.1	16.0
WRAK	2.6	74.9	2.3	1.7	0.0	21.1
BGAH	6.9	77.8	2.8	1.6	0.0	17.8
BGAK	2.3	77.2	2.4	2.0	0.0	18.4

^1^ dry-ash-free basis; ^2^ calculated by difference; method error ≤ 0.3%.

**Table 2:** Acidic-basic character of the activated carbon surfaces.

Sample	Acidic Groups Content [mmol/g]	Basic Groups Content [mmol/g]	Total Content of Surface Groups [mmol/g]	pH of Aqueous Extracts
CDAH	1.25	0.07	1.32	3.5
CDAK	1.28	0.14	1.42	5.3
WRAH	0.94	0.22	1.16	4.0
WRAK	1.34	0.44	1.78	6.7
BGAH	1.18	0.17	1.35	3.6
BGAK	1.32	0.25	1.57	5.4

**Table 3:** Textural parameters of the activated carbons prepared from waste food products.

Sample	Total ^1^		Micropore		Micropore Contribution [%]	Mean Pore Size [nm]
	Surface Area [m^2^/g]	Pore Volume [cm^3^/g]	Area [m^2^/g]	Volume [cm^3^/g]		
CDAH	993	0.893	668	0.360	40	3.596
CDAK	318	0.264	281	0.154	58	3.323
WRAH	1033	0.811	757	0.420	52	3.143
WRAK	472	0.342	431	0.237	69	2.896
BGAH	730	0.653	540	0.296	45	3.483
BGAK	445	0.343	415	0.227	66	3.076

^1^ method error in the range from 2 to 5%.

**Table 4:** Adsorption capacities toward iodine for various carbonaceous adsorbents.

Carbonaceous Adsorbent	Maximum Adsorbed Amount [mg/g]	Reference
Activated carbon from prickly pear seed cake	2527	[35]
Activated carbon from food-processing wastes—walnut shells and jujube seeds	1450 and 1200	[36]
Activated carbon from shea butter husk	1244	[37]
Activated carbon obtained from desert plant Salsolavermiculata	950	[38]
Activated carbon from spinach leaves	909	[39]
Activated carbon from household waste foods (mix of orange peels, banana peels, walnut shells, olive stones)	742	[40]
Commercial activated carbon from peat—Norit SX2	800	-
Commercial activated carbon from bituminous coal—Filtrasorb 300	900	-
Activated carbon from white rice and solid residue of “cola-type” drink	1180 and 1137	This study

**Table 5:** Langmuir and Freundlich parameters of the isotherms of methylene blue adsorption on the activated carbons prepared from waste food products.

Sample	Langmuir Model			Freundlich Model		
	q_max_	R^2^	K_L_	1/n	R^2^	K_F_
CDAH	221.3	0.9918	4.50	0.186	0.9334	141.90
CDAK	107.5	0.9812	0.20	0.480	0.9529	19.18
WRAH	188.7	0.9985	10.60	0.096	0.9814	150.04
WRAK	131.6	0.9753	1.85	0.276	0.9048	60.45
BGAH	185.2	0.9827	1.89	0.243	0.9793	101.84
BGAK	129.9	0.9703	0.94	0.374	0.9542	43.43

q_max_—the maximum adsorption capacity (mg/g); K_L_—the Langmuir adsorption equilibrium constant (dm^3^/mg); K_F_—the Freundlich equilibrium constant [mg/g (mg/dm^3^)^1/n^]; 1/n—the intensity of adsorption; R^2^—the correlation coefficients.

**Table 6:** Adsorption capacities toward methylene blue for various carbonaceous adsorbents.

Carbonaceous Adsorbent	Maximum Adsorbed Amount [mg/g]	Reference
Activated carbon from prickly pear seed cake	397	[35]
Activated carbon obtained from desert plant Salsolavermiculata	200	[38]
Activated carbon from winemaking wastes: bagasse and cluster stalks	714–847	[41]
Activated carbon from expired beverage	405	[42]
Activated carbon from molasses	370	[43]
Activated carbon from spent coffee grounds	179	[44]
Activated carbon from baobab fruit shell	114	[45]
Commercial activated carbon from peat—Norit SX2	161	[46]
Activated carbons from waste food products	108–221	This study

## Data Availability

Data are included in this paper.
